# Nonlinear association between metabolic score for insulin resistance (METS-IR) and prevalent hypertension: a cross-sectional study

**DOI:** 10.1186/s12872-026-06136-6

**Published:** 2026-06-17

**Authors:** Yan Jiang, Wei Qin

**Affiliations:** 1 Department of Cardiology, Affiliated Hospital of Nantong University, Nantong, Jiangsu 226000 China; 2 Department of cardiovascular surgery, Affiliated Hospital of Nantong University, Nantong, Jiangsu 226000 China

**Keywords:** METS‑IR, Insulin resistance, Hypertension, Nonlinear association, Threshold effect, Cross‑sectional study

## Abstract

**Background:**

Insulin resistance contributes to hypertension, but the relationship with the novel non-insulin-based metabolic score for insulin resistance (METS‑IR) remains unclear, particularly regarding potential nonlinear patterns.

**Methods:**

This cross‑sectional study included 1,592 participants. Associations between METS‑IR (continuous and tertiles) and prevalent hypertension were evaluated using logistic regression, restricted cubic splines, and two‑piecewise regression, adjusting for age, sex, lifestyle factors, diabetes, and NAFLD. Subgroup analyses examined effect modification.

**Results:**

Prevalent hypertension was present in 943 (59.2%). Each 1-unit increase in METS-IR was associated with higher hypertension odds (OR 1.06, 95% CI 1.04–1.08). A nonlinear relationship with an inflection point at 37.58 was identified. Below the inflection point (37.58), each 1-unit increase in METS-IR was associated with a 16.7% higher odds of hypertension (OR 1.167, 95% CI 1.082–1.259); above this point, the association was attenuated but remained significant (OR 1.038, 95% CI 1.007–1.069). Significant interactions were found for sex, BMI, diabetes, and NAFLD.

**Conclusions:**

METS‑IR is independently and nonlinearly associated with prevalent hypertension, exhibiting a clear threshold effect. It may serve as a practical research indicator, particularly in individuals without overt metabolic disease, though causal inference requires prospective validation.

**Supplementary Information:**

The online version contains supplementary material available at 10.1186/s12872-026-06136-6.

## Introduction

Hypertension is a major modifiable risk factor for cardiovascular disease, stroke, chronic kidney disease, and premature death globally [1]. Contemporary guidelines continue to emphasize the importance of early identification and management of elevated blood pressure to reduce long-term cardiovascular risk [2]. Increasing evidence indicates that hypertension is closely linked to metabolic dysfunction, particularly insulin resistance (IR). IR may promote blood pressure elevation through multiple interrelated mechanisms, including sympathetic nervous system overactivity, renal sodium retention, endothelial dysfunction, oxidative stress, and dysregulation of the renin-angiotensin-aldosterone system [3, 4]. Therefore, simple and reliable surrogate markers of IR may be valuable for identifying individuals with an unfavorable metabolic profile associated with hypertension.

The metabolic score for insulin resistance (METS-IR), first proposed by Bello-Chavolla et al., is a non-insulin-based index derived from fasting glucose, triglycerides, high-density lipoprotein cholesterol, and body mass index [5]. Because it integrates abnormalities in glucose metabolism, lipid metabolism, and adiposity, METS-IR has been increasingly used as a practical indicator of cardiometabolic dysfunction. Previous studies have shown that METS-IR is associated with prehypertension even in normoglycemic Chinese and Middle Eastern populations [6, 7]. Furthermore, accumulating evidence suggests that elevated METS-IR is related to hypertension across different populations and study settings [8–11]. In addition, METS-IR has been linked to other adverse cardiometabolic outcomes, including all-cause and cardiovascular mortality, suggesting that this index may capture a broader spectrum of metabolic risk beyond blood pressure alone [12].

Despite these findings, several important questions remain unresolved. First, the association between METS-IR and prevalent hypertension has not been comprehensively characterized across populations. Second, although some recent studies have suggested that this association may be nonlinear, the shape of the dose-response relationship and the potential presence of threshold effects have not been adequately elucidated [9, 10]. Third, whether the association differs according to clinically relevant characteristics, such as sex, body mass index, diabetes, and nonalcoholic fatty liver disease (NAFLD), is still unclear. Addressing these issues could enhance our understanding of the clinical utility of METS‑IR in hypertension‑related risk stratification.

It remains unclear whether the association between METS‑IR and hypertension is consistent across different demographic and metabolic subgroups. Therefore, in the present cross-sectional study, we aimed to investigate the association between METS-IR and prevalent hypertension, to examine the potential nonlinear dose-response relationship, and to explore whether this association differs across important demographic and metabolic subgroups.

## Methods

### Data source and ethical approval

This cross‑sectional study used de‑identified data from the Dryad Digital Repository (DOI: 10.5061/dryad.7d7wm3809), originally collected as part of a health examination program at Wuhan Union Hospital (January 2020 - November 2021; described by Yan et al. [13]). The original study was approved by the IRB of Tongji Medical College (Approval No. S155), and informed consent was waived due to the retrospective, anonymized nature of the data analysis. All biochemical parameters had been measured at the time of the original health examination; no additional laboratory tests were performed for this secondary analysis.

### Study population

A total of 1,830 participants aged 40–79 years who voluntarily underwent body composition analysis and liver ultrasonography were initially enrolled. Participants were excluded if they reported excessive alcohol consumption (defined as > 210 g/week for men and > 140 g/week for women), had a history of viral, autoimmune, or drug‑induced liver disease, acute illness, renal insufficiency (estimated glomerular filtration rate < 60 mL/min/1.73 m²), active malignancy, or were currently using oral or injectable steroids. Individuals with missing biochemical measurements or incomplete medical history interviews were also excluded. Following these exclusions, a final cohort of 1,592 participants was included in the present analysis.

### Definition of METS‑IR and hypertension

The Metabolic Score for Insulin Resistance (METS‑IR) was calculated using the formula established by Bello‑Chavolla et al. [5]:

$$\mathrm{METS}-\mathrm{IR}\;=\frac{\mathrm{In}\left[\left(2\times\mathrm{Fasting}\;\mathrm{Glucose}\;\left(\mathrm{mg}/\mathrm{dL}\right)\right)+\mathrm{Triglycerides}\;\left(\mathrm{mg}/\mathrm{dL}\right)\right]\times\mathrm{BMI}\;\left(\mathrm{kg}/\mathrm m^2\right)}{\mathrm{In}\left[\mathrm{HDL}-\mathrm C\;\left(\mathrm{mg}/\mathrm{dL}\right)\right]}$$

For analysis, METS‑IR was analyzed both as a continuous variable (per 1‑unit increase) and categorized into tertiles (T1: low; T2: medium; T3: high). No universally established normal reference range for METS-IR is currently available. Therefore, METS-IR was analyzed both as a continuous variable and by tertiles according to its distribution in the present cohort.

Hypertension was defined according to the 2017 ACC/AHA guidelines as systolic blood pressure (SBP) ≥ 130 mmHg, or diastolic blood pressure (DBP) ≥ 80 mmHg, or a self-reported physician diagnosis of hypertension, or current use of antihypertensive medication [14]. Among participants with hypertension, cases were identified according to measured blood pressure, self-reported physician diagnosis, or current antihypertensive medication use. A sensitivity analysis restricted to participants meeting the measured blood pressure criterion yielded similar results to the primary analysis.

### Covariate assessment

Data on demographic characteristics, lifestyle factors, and medical history were collected via standardized questionnaires. The following covariates were included in the multivariable models: age (categorized as 40–49, 50–59, 60–69, and 70–79 years), sex (male/female), tobacco use (current or former smoker vs. never smoker), alcohol use (current or former drinker vs. non‑drinker; individuals with excessive intake defined as > 210 g/week for men and > 140 g/week for women were excluded), diabetes mellitus (DM) (self‑reported diagnosis or use of glucose‑lowering medication), and non‑alcoholic fatty liver disease (NAFLD) (diagnosed via abdominal ultrasonography after excluding viral, autoimmune, drug‑induced, and alcoholic liver disease).

Biochemical parameters, including ALT, AST, UA, TC, TG, HDL-C, LDL-C, and FBG, were measured from fasting venous blood samples using a Beckman AU5800 automated biochemical analyzer. These included alanine aminotransferase (ALT), aspartate aminotransferase (AST), uric acid (UA), total cholesterol (TC), triglycerides (TG), high‑density lipoprotein cholesterol (HDL‑C), low‑density lipoprotein cholesterol (LDL‑C), and fasting blood glucose (FBG).

### Statistical analysis

Continuous variables are presented as mean ± standard deviation (SD) for normally distributed data or median (interquartile range, IQR) for skewed data. Categorical variables are expressed as frequencies (percentages). The normality of continuous variables was assessed using the Shapiro-Wilk test and visual inspection of distributional plots. Baseline characteristics were compared across METS-IR tertiles using one-way analysis of variance (ANOVA), Kruskal–Wallis tests, or chi-square tests, as appropriate.

Multivariable logistic regression models were used to estimate odds ratios (ORs) and 95% confidence intervals (CIs) for the association between METS-IR and hypertension. Four sequential models were constructed: the crude model was unadjusted; Model 1 was adjusted for age and sex; Model 2 was further adjusted for ALT, AST, and UA; and Model 3 was additionally adjusted for tobacco use, alcohol use, DM, and NAFLD. The restricted cubic spline model was fitted with three knots at the 10th, 50th, and 90th percentiles of METS-IR, and a two-piecewise logistic regression model was used to estimate the threshold effect.

Subgroup analyses were performed to assess the robustness of the association across different demographic and clinical strata, including age group, sex, and comorbidity status. Interaction terms between METS-IR and stratification variables were tested to calculate P values for interaction. All subgroup and interaction analyses were exploratory; no adjustment for multiple comparisons was applied. All analyses were performed using R (version 3.3.2) and Free Statistics (version 2.3). A two-sided *P* < 0.05 was considered statistically significant.

## Results

### Baseline characteristics

A total of 1,592 participants were included in the analysis and were evenly distributed across the METS-IR tertiles (T1, *n* = 531; T2, *n* = 530; T3, *n* = 531). Baseline demographic characteristics differed significantly among the three groups. The age distribution varied across tertiles (*P* < 0.001), with individuals aged 50–59 years accounting for the largest proportion of the study population. Sex distribution also differed markedly across tertiles (*P* < 0.001): the proportion of men increased progressively from 51.6% in T1 to 77.4% in T2 and 87.4% in T3, whereas the proportion of women decreased correspondingly. In addition, tobacco use and alcohol consumption were both more common in the higher METS-IR tertiles (*P* < 0.001) (Table 1).

**Table 1:** Baseline Characteristics of Study Participants Stratified by Tertiles of METS‑IR

Variables	Total	METS‑IR			*P* value
		T1	T2	T3	
Participants	1592	531	530	531	
Age group, n (%)					< 0.001
40–49	354 (22.2)	129 (24.3)	92 (17.4)	133 (25)	
50–59	709 (44.5)	221 (41.6)	233 (44)	255 (48)	
60–69	360 (22.6)	122 (23)	129 (24.3)	109 (20.5)	
70–79	169 (10.6)	59 (11.1)	76 (14.3)	34 (6.4)	
Gender, n (%)					< 0.001
Female	444 (27.9)	257 (48.4)	120 (22.6)	67 (12.6)	
Male	1148 (72.1)	274 (51.6)	410 (77.4)	464 (87.4)	
SBP (mmHg)	130.9 ± 15.8	127.5 ± 15.7	132.2 ± 16.1	133.1 ± 15.1	< 0.001
DBP (mmHg)	81.6 ± 11.2	78.9 ± 11.2	81.7 ± 11.0	84.3 ± 10.7	< 0.001
BMI (kg/m2)	25.4 ± 2.9	22.9 ± 1.6	25.2 ± 1.5	28.1 ± 2.7	< 0.001
ALT (U/L)	21.0 (15.0, 30.0)	17.0 (12.0, 23.0)	22.0 (16.0, 29.0)	27.0 (19.0, 39.0)	< 0.001
AST (U/L)	23.1 ± 11.0	22.0 ± 12.3	22.1 ± 9.0	25.2 ± 11.1	< 0.001
UA (µmol/L)	366.8 ± 95.8	325.4 ± 82.6	369.2 ± 88.3	405.7 ± 98.7	< 0.001
FBG (mmol/L)	5.5 ± 1.7	5.0 ± 0.9	5.5 ± 1.6	6.2 ± 2.0	< 0.001
TC (mmol/L)	4.5 ± 1.1	4.6 ± 1.1	4.3 ± 1.0	4.5 ± 1.1	< 0.001
TG (mmol/L)	1.4 (1.0, 2.2)	1.0 (0.8, 1.3)	1.4 (1.0, 1.9)	2.3 (1.6, 3.6)	< 0.001
HDL-C (mmol/L)	1.1 ± 0.3	1.4 ± 0.3	1.1 ± 0.2	0.9 ± 0.2	< 0.001
LDL-C (mmol/L)	2.7 ± 0.9	2.7 ± 0.9	2.7 ± 0.9	2.6 ± 0.9	< 0.001
Tobacco use, n (%)					< 0.001
No	1054 (66.2)	418 (78.7)	344 (64.9)	292 (55)	
Yes	538 (33.8)	113 (21.3)	186 (35.1)	239 (45)	
Alcohol.use, n (%)					< 0.001
No	1072 (67.3)	412 (77.6)	354 (66.8)	306 (57.6)	
Yes	520 (32.7)	119 (22.4)	176 (33.2)	225 (42.4)	
Hypertension, n (%)					< 0.001
No	649 (40.8)	304 (57.3)	190 (35.8)	155 (29.2)	
Yes	943 (59.2)	227 (42.7)	340 (64.2)	376 (70.8)	
Diabetes, n (%)					< 0.001
No	1094 (68.7)	445 (83.8)	363 (68.5)	286 (53.9)	
Yes	498 (31.3)	86 (16.2)	167 (31.5)	245 (46.1)	
NAFLD, n (%)					< 0.001
No	619 (38.9)	349 (65.7)	189 (35.7)	81 (15.3)	
Yes	973 (61.1)	182 (34.3)	341 (64.3)	450 (84.7)	

Data are mean ± SD, median (IQR), or n (%). P values were derived from one-way ANOVA, Kruskal-Wallis test, or chi-square test, as appropriate. T1, T2, and T3 denote the first, second, and third tertiles of METS‑IR. *P < 0.05, **P < 0.01, and ***P < 0.001

*Abbreviations:* *ALT* Alanine aminotransferase, *AST* Aspartate aminotransferase, *BMI* Body mass index, *DBP* Diastolic blood pressure, *FBG* Fasting blood glucose, *HDL-C* High-density lipoprotein cholesterol, *IQR* Interquartile range, *LDL-C* Low-density lipoprotein cholesterol, *METS-IR* Metabolic score for insulin resistance, *NAFLD* Non-alcoholic fatty liver disease, *SBP* Systolic blood pressure, *SD* Standard deviation, *TC* Total cholesterol, *TG* Triglyceride, *UA* Uric acid

Clinical and laboratory parameters also differed significantly across METS-IR tertiles. SBP, DBP, and BMI increased progressively from T1 to T3 (*P* < 0.001). Similarly, ALT, AST, uric acid, fasting blood glucose, triglycerides, total cholesterol and LDL-C differed significantly among the three groups (*P* < 0.001), whereas HDL-C levels decreased with increasing METS-IR (*P* < 0.001). The prevalence of hypertension, diabetes, and NAFLD also increased significantly across tertiles (*P* < 0.001). Of the 943 participants with hypertension, 62% were identified by measured blood pressure alone, 18% by self‑reported physician diagnosis, and 20% by current antihypertensive medication use (categories were not mutually exclusive).

### Association Between METS-IR and hypertension

**Fig. 1:**
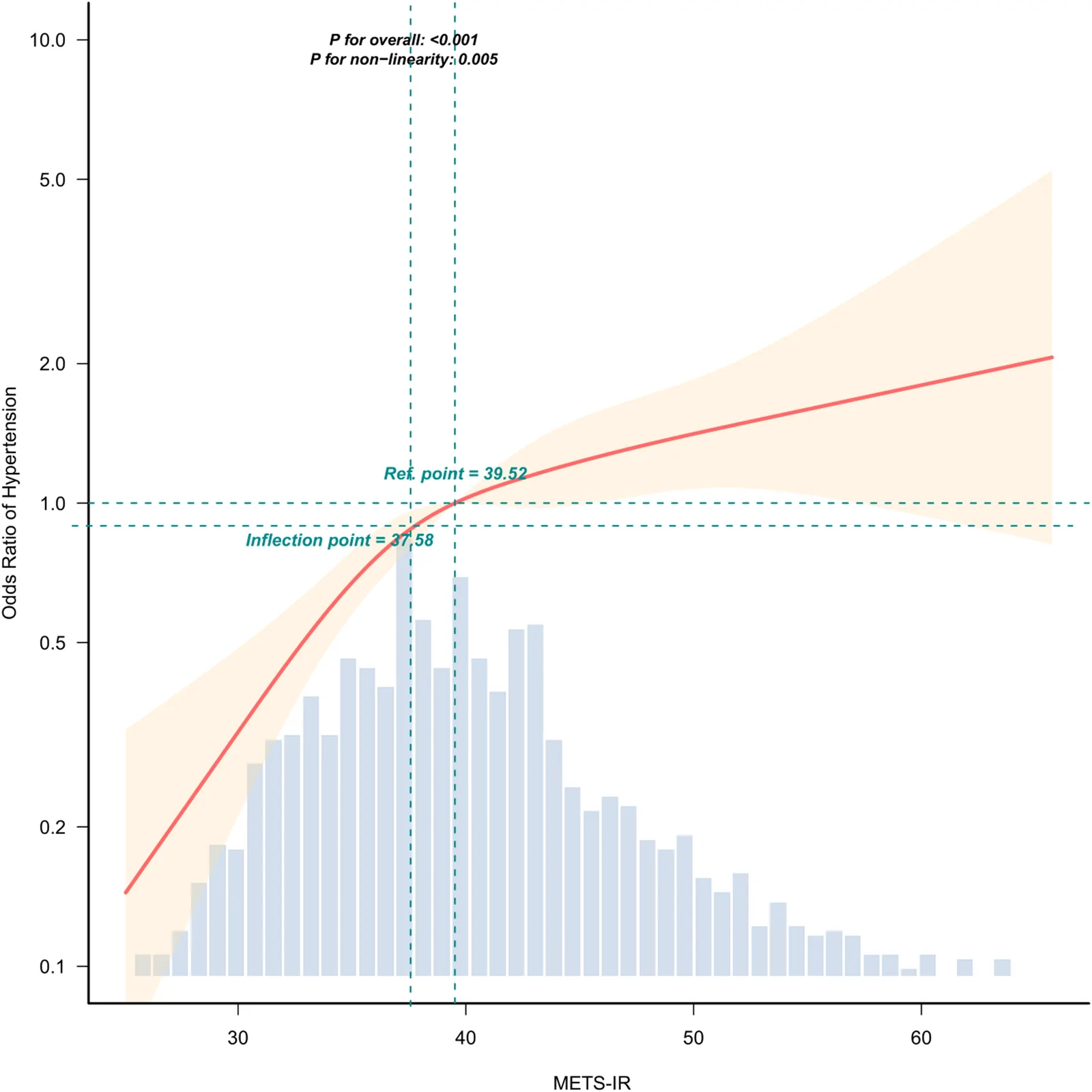
Restricted cubic spline analysis of the association between METS-IR and prevalent hypertension. The red solid line represents the estimated odds ratio, and the shaded blue area represents the 95% confidence interval. The vertical dashed line indicates the estimated inflection point at METS-IR = 37.58. The model was adjusted for age, sex, ALT, AST, UA, tobacco use, alcohol use, DM, and NAFLD

Restricted cubic spline analysis revealed a significant nonlinear dose-response association between METS-IR and prevalent hypertension. After adjustment for age, sex, ALT, AST, UA, tobacco use, alcohol use, diabetes mellitus, and NAFLD, the overall association was statistically significant (P for overall < 0.001), and a nonlinear pattern was also observed (P for nonlinearity = 0.005). The curve rose more steeply at lower METS-IR levels and continued to increase more gradually at higher levels, whereas the 95% confidence intervals widened at both ends of the distribution, particularly in the upper range (Fig. 1).

In the logistic regression analyses, METS-IR was significantly associated with hypertension when modeled as both a continuous variable and tertiles (Table 2). In the continuous analysis, each 1-unit increase in METS-IR was associated with higher odds of hypertension in the crude model (OR 1.09, 95% CI 1.07–1.10; *P* < 0.001), and this association remained statistically significant after sequential adjustment for potential confounders. In the fully adjusted model, the OR was 1.06 (95% CI 1.04–1.08; *P* < 0.001). When METS-IR was categorized into tertiles, participants in T2 and T3 had significantly higher odds of hypertension than those in T1 across all models. In Model 3, the ORs were 1.66 (95% CI 1.24–2.21; *P* = 0.001) for T2 and 2.03 (95% CI 1.46–2.81; *P* < 0.001) for T3, and the trend across tertiles remained statistically significant in all models (P for trend < 0.001).

**Table 2:** Multivariable-adjusted associations between METS-IR and the prevalence of hypertension

Variable	Crude model		Model 1		Model 2		Model 3	
	OR (95%CI)	*P* value	OR (95%CI)	*P* value	OR (95%CI)	*P* value	OR (95%CI)	*P* value
METS‑IR (continuous)	1.09 (1.07–1.10)	< 0.001	1.09 (1.07–1.10)	< 0.001	1.08 (1.06–1.1)	< 0.001	1.06 (1.04–1.08)	< 0.001
METS‑IR (tertiles)								
T1	1(Ref)		1(Ref)		1(Ref)		1(Ref)	
T2	2.40 (1.87–3.07)	< 0.001	2.06 (1.57–2.69)	< 0.001	1.94 (1.47–2.55)	< 0.001	1.66 (1.24–2.21)	0.001
T3	3.25 (2.52–4.19)	< 0.001	3.26 (2.45–4.33)	< 0.001	2.84 (2.10–3.83)	< 0.001	2.03 (1.46–2.81)	< 0.001
*P* for trend		< 0.001		< 0.001		< 0.001		< 0.001

Values are presented as odds ratio (OR) with 95% confidence interval (CI). *P < 0.05, **P < 0.01, and ***P < 0.001

Crude Model: unadjusted. Model 1: adjusted for age and sex. Model 2: additionally adjusted for ALT, AST, and UA. Model 3: additionally adjusted for tobacco use, alcohol use, DM, and NAFLD

*Abbreviations:* *ALT* Alanine aminotransferase, *AST* Aspartate aminotransferase, *CI* Confidence interval, *METS‑IR* Metabolic score for insulin resistance, *NAFLD* Non‑alcoholic fatty liver disease, *OR* Odds ratio, *Ref* Reference, *UA* Uric acid

Threshold effect analysis further showed that the magnitude of the association differed across the identified inflection point of 37.58 (Table 3). Below the estimated inflection point of 37.58, each 1-unit increase in METS-IR was associated with a larger increase in the odds of hypertension; above this point, the association was attenuated yet remained statistically significant (OR 1.038, 95% CI 1.007–1.069; *P* = 0.015). The test for nonlinearity was statistically significant (P for nonlinearity = 0.004).

**Table 3:** Threshold effect analysis of the association between METS‑IR and hypertension

METS-IR	OR (95% CI)	*P* value
< 37.58	1.167 (1.082–1.259)	< 0.001
≥ 37.58	1.038 (1.007–1.069)	0.015
*P* for non-linearity		0.004

ORs are reported per 1‑unit increase in METS‑IR within each interval. The model was adjusted for age, sex, ALT, AST, UA, tobacco use, alcohol use, DM, and NAFLD (Model 3). The inflection point (37.58) was identified using a restricted cubic spline model.P for non-linearity was derived from the likelihood ratio test comparing the spline model to a linear model. Abbreviations: CI, confidence interval; METS-IR, metabolic score for insulin resistance; OR, odds ratio. *P < 0.05, **P < 0.01, and ***P < 0.001

### Sensitivity analyses

**Fig. 2:**
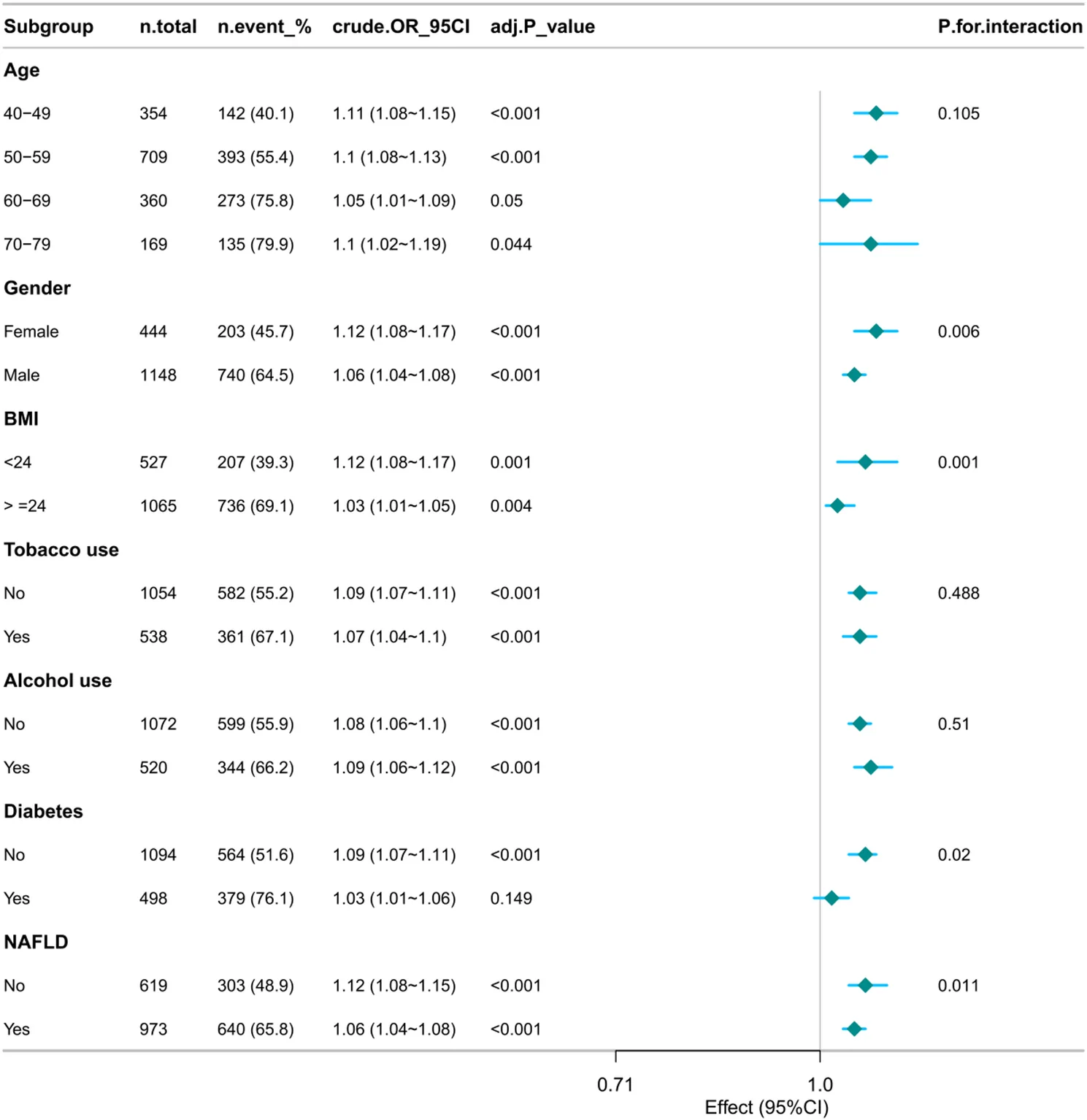
Subgroup analysis of the association between METS‑IR and hypertension. Odds ratios (ORs) and 95% confidence intervals (CIs) were estimated using multivariable logistic regression Model 3 adjusted for all covariates except the stratification variable. P values for interaction are presented on the right side of the plot

In subgroup analyses based on the fully adjusted model, the positive association between METS-IR and hypertension was observed across all examined strata, including age, sex, BMI, tobacco use, alcohol use, diabetes, and NAFLD (Fig. 2). No significant interaction was detected for age, tobacco use, or alcohol use. Significant interactions were found for sex, BMI, diabetes, and NAFLD: the association was stronger in women than in men (P for interaction = 0.006), in participants with BMI < 24 kg/m² than in those with BMI ≥ 24 kg/m² (*P* = 0.001), in participants without diabetes than in those with diabetes (*P* = 0.020), and in participants without NAFLD than in those with NAFLD (*P* = 0.011).

Variance inflation factors for all variables in the fully adjusted model were below 5. In a sensitivity analysis excluding diabetes and NAFLD from the model, the OR for METS-IR and prevalent hypertension was 1.05 (95% CI 1.03–1.07). Excluding participants aged ≥ 70 years (*n* = 169) yielded an OR of 1.05 (95% CI 1.03–1.07). Restricting the outcome definition to measured blood pressure alone produced an OR of 1.06 (95% CI 1.04–1.08).

## Discussion

In this cross-sectional study of 1,592 participants, higher METS-IR was independently associated with greater odds of prevalent hypertension. This association remained significant when METS-IR was analyzed both as a continuous variable and by tertiles, indicating that the finding was robust to different modeling strategies. More importantly, we identified a significant nonlinear dose-response relationship, with a steeper increase in the odds of hypertension at lower METS-IR levels and a more gradual increase beyond the inflection point. Subgroup analyses further suggested that the association was more pronounced in women, individuals with BMI < 24 kg/m², and those without diabetes or NAFLD. Taken together, these findings support METS-IR as a practical marker of metabolic dysfunction associated with hypertension.

Our findings are broadly consistent with previous studies showing that elevated METS-IR is associated with a higher likelihood of hypertension across different populations. A meta-analysis including more than 300,000 participants reported that individuals in the highest METS-IR category had substantially higher odds or likelihood of hypertension than those in the lowest category [15]. Similar positive associations have been observed in normal-weight Chinese adults [16], non-overweight adults in a Northeastern Chinese prospective cohort [8], Japanese normoglycemic populations [17], and NHANES-based analyses [9, 18]. Recent studies have further suggested that the relationship between METS-IR and hypertension may not be strictly linear [9, 10, 18]. In addition, evidence from specific subgroups, including military young adults and Middle Eastern populations, supports the robustness of this association across different demographic and metabolic settings [19, 20]. Beyond hypertension itself, METS-IR has also been linked to related cardiometabolic phenotypes and outcomes, including nonalcoholic fatty liver disease, arterial stiffness, stroke among patients with hypertension, and adverse cardiovascular prognosis [21–25]. This association with NAFLD has also been supported in a nonobese Chinese population, in which a dose-response relationship between METS-IR and NAFLD was observed, suggesting that the metabolic relevance of METS-IR extends beyond overt obesity [24]. Moreover, Mendelian randomization evidence supports a potentially causal role of insulin resistance in hypertension and cardiovascular disease [26]. In line with these reports, our study adds evidence from a hospital-based Chinese population and further demonstrates a threshold effect, suggesting that the adverse association of METS-IR with hypertension may be particularly evident within a lower-to-moderate range of the index, which may help identify individuals at risk before metabolic decompensation becomes clinically overt.

Several biological mechanisms may explain the observed association. METS-IR reflects disturbances in glucose metabolism, lipid metabolism, and adiposity, all of which are closely related to insulin resistance. Insulin resistance contributes to elevated blood pressure through multiple pathways, including sympathetic nervous system overactivation, renal sodium retention, endothelial dysfunction, oxidative stress, and dysregulation of the renin–angiotensin–aldosterone system [4, 27]. In addition, METS-IR has been associated with arterial stiffness and other subclinical vascular changes, which may represent an intermediate link between metabolic dysfunction and hypertension development [21, 23]. Therefore, METS-IR may not only serve as a surrogate index of insulin resistance, but also capture a broader cardiometabolic profile relevant to blood pressure regulation. Insulin resistance may influence blood pressure through disturbances in lipid metabolism and endothelial function. In endothelial cells, insulin activates the PI3K/Akt/eNOS pathway, increasing nitric oxide bioavailability and promoting vasodilation. In insulin-resistant states, this pathway is impaired, favoring pro-inflammatory and vasoconstrictive signaling, which contributes to endothelial dysfunction, arterial stiffness, and elevated blood pressure [28, 29]. Additionally, improved insulin sensitivity upregulates hepatic LDL receptor expression, lowering circulating LDL-C and potentially reducing endothelial stress in the vasculature and kidneys, thereby aiding blood pressure reduction. Thus, the association observed in our study may reflect not only systemic insulin resistance but also related disturbances in vascular endothelial homeostasis.

An interesting finding of the present study is the heterogeneity observed across subgroups. The association appeared stronger in women, participants with BMI < 24 kg/m², and those without diabetes or NAFLD. One possible explanation is that in individuals without overt metabolic disease, a higher METS-IR may better identify an early but clinically meaningful disturbance in insulin sensitivity, whereas in those with established diabetes or NAFLD, the effect of METS-IR may be partly attenuated by competing metabolic abnormalities or ongoing treatment. The stronger association in women may reflect sex-related differences in fat distribution, hormonal status, vascular function, and susceptibility to metabolic stress. Likewise, the stronger association in non-overweight individuals suggests that METS-IR may help identify hypertension-related metabolic risk even in people who would not traditionally be considered high risk based on body size alone. These interpretations, however, remain speculative and should be confirmed in future mechanistic and longitudinal studies.

This study has several strengths. First, we evaluated METS-IR using both categorical and continuous approaches, which improved the robustness of the findings. Second, by combining restricted cubic spline analysis with threshold-effect modeling, we were able to characterize the dose-response pattern more comprehensively than would be possible with conventional linear regression alone. Third, the subgroup analyses allowed us to explore potential effect modification in clinically relevant strata. Together, these analytical strategies provide a more nuanced understanding of the relationship between METS-IR and prevalent hypertension.

### Limitations

Several limitations should be acknowledged. First, due to the cross-sectional design, causality cannot be inferred, and reverse causation cannot be excluded. Second, although age was adjusted as a categorical covariate, the wide age range (40–79 years) may still introduce residual confounding. Third, residual confounding remains possible from unmeasured factors (e.g., physical activity, dietary sodium, socioeconomic status, menopausal status), which would generally tend to overestimate the association; therefore, our ORs should be interpreted conservatively. Fourth, METS-IR is an indirect surrogate of insulin resistance, without direct measures like the hyperinsulinemic-euglycemic clamp. Fifth, the identified inflection point (37.58) may be population-specific and derived from the same dataset, requiring external validation. Sixth, the single-center health examination cohort may introduce selection bias, limiting generalizability to other populations. Seventh, baseline imbalance across METS-IR tertiles (overrepresentation of men in the highest tertile) may affect the stability of subgroup analyses, which should be interpreted cautiously. Eighth, the composite definition of hypertension may introduce misclassification, and detailed information on antihypertensive medication (classes, duration, control status) was unavailable, potentially leading to residual confounding. Future studies using repeated standardized blood pressure measurements and comprehensive medication data are warranted.

## Conclusions

In conclusion, higher METS-IR was independently associated with greater odds of prevalent hypertension, and this association followed a nonlinear pattern. METS-IR may be a simple and potentially useful indicator in research settings, pending external validation. Prospective multicenter studies are needed to confirm the observed dose-response relationship, validate the threshold effect, and clarify the mechanisms linking METS-IR to hypertension.

## Supplementary Information

Supplementary Material 1.

## Data Availability

The data that support the findings of this study are openly available in the Dryad Digital Repository at https://doi.org/10.5061/dryad.7d7wm3809. This dataset was originally generated by Yan et al. 13 and includes clinical and biochemical data from 1,592 participants who underwent health examinations at Wuhan Union Hospital between January 2020 and November 2021. The datasets used and/or analyzed during the current study are also available from the corresponding author upon reasonable request.
