# Supplementary material for: Nonlinear association between metabolic score for insulin resistance (METS-IR) and prevalent hypertension: a cross-sectional study
Source: BMC Cardiovasc Disord. 2026 Jun 17;26:740. doi: 10.1186/s12872-026-06136-6 (PMC13508455; doi:10.1186/s12872-026-06136-6)
Supplement: Supplementary file 1 [file 12872_2026_6136_MOESM1_ESM.docx]

Supplementary Figure S1

Flow chart of the study.
